# Isotonic Glucose Injections for Postherpetic Neuralgia in the Elderly

**DOI:** 10.7759/cureus.29740

**Published:** 2022-09-29

**Authors:** Jan Kersschot, Ilan Karavani

**Affiliations:** 1 Family Medicine, No Primary Affiliation, Antwerp, BEL; 2 Dermatology, No Primary Affiliation, Antwerp, BEL

**Keywords:** atp, glucose 5%, dextrose prolotherapy, dextrose 5%, intradermal injection, postherpetic neuralgia, isotonic glucose injections

## Abstract

Postherpetic neuralgia (PHN) is a painful condition which is difficult to treat, especially among the elderly. This clinical case describes the treatment of an 88-year-old patient with PHN who continued to suffer from pain for several months despite oral and transdermal pain treatment. Multiple intradermal glucose 5% injections allowed her to discontinue her pain medication regimen after four sessions. The improvement was sustained at the four-month follow-up after the last procedure. A fifth session was performed because of a flaring up of the pain.

## Introduction

Primary care providers often encounter immunocompromised or elderly patients with herpes zoster associated with severe neuropathic pain. Postherpetic neuralgia (PHN) is defined as a pain that persists for three months or longer after skin restitution. It is the most common complication of herpes zoster. Older patients in particular continue to experience intermittent neuropathic symptoms, including allodynia and pain. The pain may be burning or stabbing. Current treatment includes anticonvulsants, opioids, systemic tricyclic antidepressants, topical lidocaine, capsaicin, film-forming bupivacaine, intradermal botulinum toxin injections, nerve blocks with local anesthetics, and nerve stimulation.

The PHN is the most common long-term complication of varicella-zoster virus reactivation. Typically, the neuropathic pain presents in a unilateral dermatome pattern. It can persist for several months after the onset of the skin rash. It is rather difficult to manage, and can cause serious morbidity, especially in patients older than 60. Structural changes and molecular signaling underlie the sensitization of nociceptive pathways, which include alteration in ion channels, activation of immune cells, glial mediators, and epigenetic regulation [1].

Most patients with PHN overuse pain medications. One of the goals of isotonic glucose injections as described in this clinical case report is to reduce the use of pain medications. The treatment consists of a series of intradermal injections of glucose 5% into the pain region. Diagnosis is based on history. As a result, additional investigations such as ultrasound or lab test are not required. Unlike prolotherapy [2], hypertonic glucose solutions such as glucose 10%-20% are not used. Adding local anesthetics is not required.

## Case presentation

An 88-year-old lady (born July 1934) came to the clinic for pain which started on May 5, 2021. She complained about severe flank pain on the left side. To exclude kidney stones, she received an ultrasound of the urinary tract which came out normal. Acetaminophen and tradonal gave no pain relief. A tiny skin lesion (less than half a centimeter diameter or 0.2 in) was identified by the doctor in the hospital as the remains of a herpes vesicle. There is no photograph available of that skin lesion. There was no test that confirmed that the skin lesion was actually a Herpes Viral infection. She was treated with acyclovir but the pain in the left flank remained. While she stayed in the hospital, she was also observed by a physician in Physical Therapy and Rehabilitation because she also complained about pain in both legs. MRI showed spinal stenosis on the lumbar level. She received several epidural steroid injections for the pain in the legs. The pain in the legs persisted after the epidural injections. As she continued to suffer for months from intermittent neuropathic symptoms in the left flank, on the lateral side of dermatome T11-T12, she wanted a second opinion. Her complaints were resistant to painkillers and lidocaine transdermal patches. Unfortunately, other treatment options such as paravertebral blocks with local anesthetics or deep perineural glucose 5% injections were not applied during her stay in the hospital.

**Figure 1:**
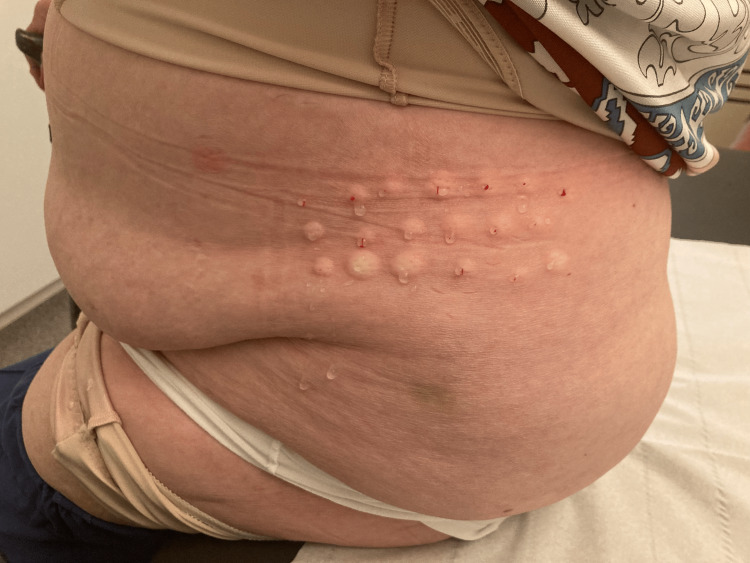
Multiple intracutaneous injections in the pain region in the left flank.

**Figure 2:**
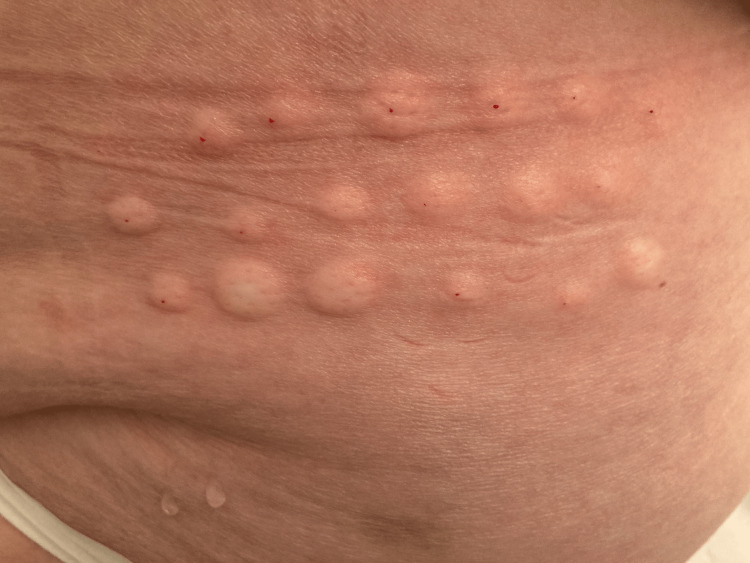
Multiple intracutaneous injections (after cleaning the injected area).

The first time I saw the patient for this problem (Feb 25, 2022), she explained that she complained for several months in a row about intermittent pain in the left flank which was resistant to pain medication, including tramadol and gabapentin. At that point of time, no photographs of the region were taken because the skin looked entirely normal. As she had already had good results after a series of isotonic glucose injections for osteoarthritis in her knees, she asked if she could have a local treatment with isotonic glucose for her neuropathic pain in the left flank as well. The treatment was based on the location of the pain region as indicated by the patient during her first visit. After skin disinfection with chlorhexidine in alcohol, a syringe with 5 mL glucose 5% in water (G5W) and a 30G 1/2 in needle were used to give multiple injections in the region as indicated by the patient. She received about 20 intradermal injections with G5W on the following dates: Feb 25, March 3, March 10 and April 14, 2022. The total volume injected each session was about 5 mL. The injections were given intracutaneously. After each session, her neuralgic pain improved considerably. The improvement lasted longer with each session. Assessment of the pain with, for example, a visual analog scale (VAS) score was not applied. She said that after these four sessions her pain had disappeared and that she had stopped taking her pain medication. During a check-up on July 20, she mentioned that occasionally she felt a small sting in the area but no more pain. A month later (August 23), she said the pain had flared up again after an emotional conflict. She, again, received intradermal injections with glucose 5%. As in the previous sessions, about 20 injections were given in the painful region (see Figure 1). After wiping clean the skin after the injections, one can see that the injections were given intracutaneously and not subcutaneously (see Figure 2). She was asked by email about the clinical evolution of the pain, and she replied on August 25 that the pain had disappeared completely the next day without taking any pain medication.

## Discussion

Injections of isotonic glucose (or dextrose) have been used, for example, to treat carpal tunnel syndrome [3], epidural injections [4], and nerve hydrodissection [5]. The exact mechanism of action of isotonic glucose injections is likely multifactorial. Regulation of neural inflammation and upregulation of pro-inflammatory cytokines are described as effects of glucose injections [6]. Patients with PHN probably have irritated dermal nociceptors which show spontaneous activity in the absence of stimulation [7]. The energy requirement of the nervous system is primarily met by glucose which is oxidized via glycolysis and oxidative phosphorylation to produce ATP [8]. It has been shown that ATP injection can increase the expression of markers of regeneration in sensory neurons, such as phospho-STAT3 and GAP43 [9]. It has also been found that ATP infusion improves pain and allodynia in patients with PHN [10-11]. The question of whether regional intradermal glucose injections lead to increased ATP production and subsequent regeneration in peripheral sensory neurons is still subject to scientific debate. More research in this field may show that glucose also acts through miR-186-5p in neuropathic pain [12].

When reviewing the literature about isotonic glucose (or dextrose) injections, it is surprising to notice that these injections have been used for decades for a variety of musculoskeletal and mild neuropathic pain [13-18]. Although this treatment modality is obviously inexpensive and safe, it is unclear why it is still underused. Having a scientific explanation of the mode of action might be helpful, as well as some well-designed clinical studies.

## Conclusions

This article describes the use of intracutaneous injections of isotonic glucose for the treatment of postherpetic pain. PHN is quite difficult to manage, especially in the elderly. As a result, it is important that new treatment options are available for these patients. Physicians may consider the use of intradermal glucose 5% injections as an easy, safe, and inexpensive treatment option for postherpetic pain. However, no clinical trials are available yet. This article is written to invite the medical community to plan more research in this field.

## References

[1] Finnerup NB, Kuner R, Jensen TS (2021). Neuropathic pain: from mechanisms to treatment. Physiol Rev.

[2] Zhu M, Rabago D, Chung VC, Reeves KD, Wong SY, Sit RW (2022). Effects of hypertonic dextrose injection (prolotherapy) in lateral elbow tendinosis: a systematic review and meta-analysis. Arch Phys Med Rehabil.

[3] Wu YT, Ke MJ, Ho TY, Li TY, Shen YP, Chen LC (2018). Randomized double-blinded clinical trial of 5% dextrose versus triamcinolone injection for carpal tunnel syndrome patients. Ann Neurol.

[4] Maniquis-Smigel L, Dean Reeves K, Jeffrey Rosen H, Lyftogt J, Graham-Coleman C, Cheng AL, Rabago D (2017). Short term analgesic effects of 5% dextrose epidural injections for chronic low back pain: a randomized controlled trial. Anesth Pain Med.

[5] Lam KH, Hung CY, Chiang YP, Onishi K, Su DC, Clark TB, Reeves KD (2020). Ultrasound-guided nerve hydrodissection for pain management: rationale, methods, current literature, and theoretical mechanisms. J Pain Res.

[6] Wu YT, Chen YP, Lam KH, Reeves KD, Lin JA, Kuo CY (2022). Mechanism of glucose water as a neural injection: a perspective on neuroinflammation. Life (Basel).

[7] Fields HL, Rowbotham M, Baron R (1998). Postherpetic neuralgia: irritable nociceptors and deafferentation. Neurobiol Dis.

[8] Ashrafi G, Ryan TA (2017). Glucose metabolism in nerve terminals. Curr Opin Neurobiol.

[9] Wu D, Lee S, Luo J (2018). Intraneural injection of ATP stimulates regeneration of primary sensory axons in the spinal cord. J Neurosci.

[10] Moriyama M, Kitamura A, Ikezaki H, Nakanishi K, Kim C, Sakamoto A, Ogawa R (2004). Systemic ATP infusion improves spontaneous pain and tactile allodynia, but not tactile hypesthesia, in patients with postherpetic neuralgia. J Anesth.

[11] Hayashida M, Fukuda K, Fukunaga A (2005). Analgesic effect of intravenous ATP on postherpetic neuralgia in comparison with responses to intravenous ketamine and lidocaine. J Anesth.

[12] Huang A, Ji L, Huang Y, Yu Q, Li Y (2022). miR-185-5p alleviates CCI-induced neuropathic pain by repressing NLRP3 inflammasome through dual targeting MyD88 and CXCR4. Int Immunopharmacol.

[13] Kim MY, Na YM, Moon JH (1997). Comparison on treatment effects of dextrose water, saline and lidocaine for trigger point injection. J Kor Acad Rehab Med.

[14] Lyftogt J (2007). Subcutaneous prolotherapy for achilles tendinopathy: the best solution?. Austr Musculoskeletal Med J.

[15] Kersschot J (2022). Glucopuncture for rotator cuff related shoulder pain: an alternative for cortisone?. Clin Rev Cases.

[16] Mansiz-Kaplan B, Nacir B, Pervane-Vural S, Tosun-Meric O, Duyur-Cakit B, Genc H (2022). Effect of perineural dextrose injection on ulnar neuropathy at the elbow: a randomized, controlled, double-blind study. Arch Phys Med Rehabil.

[17] Kersschot J (2022). Intradermal glucose injections for mild localized neuropathic pain - a new approach to reduce pain medication. Global J Med Res.

[18] Lam SK, Reeves KD, Cheng AL (2017). Transition from deep regional blocks toward deep nerve hydrodissection in the upper body and torso: method description and results from a retrospective chart review of the analgesic effect of 5% dextrose water as the primary hydrodissection injectate to enhance safety. Biomed Res Int.

